# Application of Physiologically Based Pharmacokinetic Modeling to Evaluate the Drug–Drug and Drug–Disease Interactions of Apatinib

**DOI:** 10.3389/fphar.2021.780937

**Published:** 2021-11-22

**Authors:** Hongrui Liu, Yiqun Yu, Nan Guo, Xiaojuan Wang, Bing Han, Xiaoqiang Xiang

**Affiliations:** ^1^ Department of Pharmacy, Minhang Hospital, Fudan University, Shanghai, China; ^2^ Department of Clinical Pharmacy, School of Pharmacy, Fudan University, Shanghai, China

**Keywords:** apatinib, tyrosine kinase inhibitors (TKIs), physiologically based pharmacokinetic (PBPK) models, drug–drug interaction (DDI), drug-disease interaction (DDZI)

## Abstract

**Aim:** Apatinib is an orally administered vascular epidermal growth factor receptor (VEGFR)-tyrosine kinase inhibitors approved for the treatment of advanced gastric adenocarcinoma or gastric esophageal junction adenocarcinoma. Apatinib is predominantly metabolized by CYP3A4/5, followed by CYP2D6. The present study aimed to evaluate the potential drug–drug interaction (DDI) and drug–disease interaction (DDZI) risks of apatinib in Chinese volunteers.

**Methods:** Modeling and simulation were conducted using Simcyp Simulator. The input parameters required for modeling were obtained from literature research or experiments. Then, the developed physiologically based pharmacokinetic (PBPK) models were applied to evaluate single-dose DDI potential in Chinese healthy volunteers with weak and moderate CYP3A inhibitors, strong CYP2D6 inhibitors, as well as CYP3A4 inducers. The DDZI potential was also predicted in patients with hepatic or renal impairment.

**Results:** The developed PBPK models accurately assessed apatinib pharmacokinetics following single-dose administration in Chinese healthy volunteers and cancer patients. The DDI simulation showed 2–4-fold changes in apatinib exposures by moderate CYP3A4 inhibitors and CYP3A4 inducers. A moderate increase of apatinib exposure (1.25–2-fold) was found with strong CYP2D6 inhibitor. In the DDZI simulation with hepatic impairment, the AUC of apatinib was significantly increased by 2.25-fold and 3.04-fold for Child–Pugh B and Child–Pugh C, respectively, with slightly decreased C_max_ by 1.54 and 1.67-fold, respectively.

**Conclusion:** The PBPK models developed in the present study would be highly beneficial to quantitatively predict the pharmacokinetic changes of apatinib under different circumstances, which might be difficult to evaluate clinically, so as to avoid some risks in advance.

## Introduction

Drug–drug interaction (DDI) is a phenomenon of modification of pharmacological activity of one drug by another co-administered drug (Min and Bae, 2017; Rekić et al., 2017). Pharmacokinetic (PK) DDIs are the most common type and happens when the absorption, distribution, metabolism, or excretion (ADME) of the substrates are affected by co-administered drugs, resulting in an increased or decreased exposure of the substrates (Min and Bae, 2017; Chung and Kesisoglou, 2018; Wu et al., 2020). Understanding how certain co-administered drugs affect the exposure changes of substrate is essential for drug development and application (Zhang et al., 2012). The Japanese Medicines and Medical Devices Administration (PMDA), European Medicines Agency (EMA), and U.S. Food and Drug Administration (FDA) have issued separate DDI guidelines, emphasizing the predictive use of comprehensive mechanism methods, such as physiologically based pharmacokinetic (PBPK) models, for quantitative evaluation of potential DDI risk in different periods of drug discovery and development (Committee for Human Medicinal Products, 2012; Drug Interaction Studies, 2012; Drug Interaction Studies, 2014). Due to the fact that most drugs are primarily eliminated *via* liver and kidneys, the impairment of organ function of liver and kidney is one of the important intrinsic factors for regulating drug exposure, that is, drug–disease interaction (DDZI). Consequently, the FDA and EMA also published guidelines to assess the impact of hepatic and/or renal impairment (HIs and RIs, respectively) on the pharmacokinetics of drugs, providing the dosing and labeling recommendations (Guidance for Industry, 2003; Guidance for Industry, 2010; Committee for Human Medicinal Products, 2014; Committee for Human Medicinal Products, 2015).

Apatinib is an oral small-molecule tyrosine kinase inhibitor (TKI), which can competitively bind to the tyrosine ATP binding site in vascular endothelial growth factor receptor (VEGFR), selectively inhibit the activity of VEGFR tyrosine kinases, and block the downstream signaling, therefore suppressing the tumor angiogenesis (Roviello et al., 2016; Geng et al., 2018; Meng et al., 2020). Apatinib shows good oral bioavailability and tolerance in humans. In 2014, apatinib was approved by the National Medical Products Administration for the treatment of advanced gastric adenocarcinoma or gastric esophageal junction adenocarcinoma (Roviello et al., 2016; Geng et al., 2018). Clinical trials have shown that apatinib has certain objective remission rate and survival benefit in the treatment of a variety of malignant tumors, i.e., breast cancer, non–small cell lung cancer, hepatocellular carcinoma, and colorectal cancer (Zhang, 2015; Xue et al., 2018; Fathi Maroufi et al., 2020). *In vitro* metabolism studies showed that apatinib was the substrate of P450 enzymes, which was mainly metabolized *via* CYP3A4/5 and, to a lesser extent, by CYP2D6 (Ding et al., 2013). The plasma exposure of apatinib was reported to be significantly affected by co-administration with itraconazole (AUC_0–t_ increased by 75%) or rifampin (AUC_0–t_ decreased by 83%) in humans, indicating the high potential of DDIs by affecting drug metabolism enzymes for apatinib (Liu et al., 2018). However, the effects of moderate and weak enzyme inhibitors or inducers on the exposures of apatinib have not been evaluated since cancer patients normally receive other drugs, i.e., hypoglycemics, antihypertensive, anticoagulants, and antifungal, many of which are enzyme inhibitors or inducers. Further research is needed to evaluate its potential involvement in DDIs for the rational clinical administration. In addition, the potential risk assessments of apatinib’s pharmacokinetic changes in RIs or HIs will also be of great significance to recommend appropriate dosing regimens.

PBPK modeling is a mathematical predictive approach, which can quantitatively predict ADME process of chemical substances in human or animal by extrapolating *in vitro*, *in situ*, and *in silico* drug-dependent parameters (Min and Bae, 2017; Wu et al., 2020). Some excellent researches and reviews have illustrated the ability of PBPK modeling in drug development (Li et al., 2021; Nauwelaerts et al., 2021; Peng et al., 2021). Hence, a growing emphasis on the application of PBPK modeling has been placed to quantitatively evaluate the potential risks of DDI and DDZI of drugs *in vivo* (Ono et al., 2017; Rasool et al., 2017; Rasool et al., 2021; Rasool and Läer, 2021). Accordingly, developing a PBPK model of apatinib to quantitatively assess the influence of external and internal factors on apatinib pharmacokinetics would be of great benefit. In the present research, we aimed to establish and verify PBPK models of apatinib on the basis of the reported clinical trials, then the verified PBPK model was applied to evaluate the potential DDI risks with CYP3A4 and CYP2D6 inhibitors or inducers, as well as the DDZI outcomes in HIs and RIs. We focused on gaining insights into the rational clinical administration of apatinib by using the dynamic modeling approach.

## Materials and Methods

### Clinical Pharmacokinetic Studies of Apatinib

A systematic literature research was conducted to obtain clinical pharmacokinetic data of apatinib. Briefly, the mean plasma concentration–time profiles of apatinib were determined in Chinese advanced colorectal cancer patients (*n* = 20) at an oral dose of 750 mg following single administration (Ding et al., 2013). The reported single-dose DDI studies were conducted in Chinese healthy volunteers (*n* = 20) with co-administration of rifampin and itraconazole (Liu et al., 2018). Subjects either received 750 mg of oral apatinib mesylate alone on day 1 or treated with 600 mg of oral rifampin once daily for 10 continuous days with concomitant administration of 750 mg apatinib on day 6. For DDI studies with itraconazole, each subject received 250 mg oral apatinib mesylate alone on day 1 or 6-day repeated doses of 100 mg oral itraconazole once daily with concomitant administration of 250 mg apatinib on day 4.

### Development of a Dynamic PBPK Model for Apatinib

All simulations were conducted using a commercially available PBPK software, Simcyp Simulator (version 16; Simcyp Limited, Sheffield, UK). The input parameters for apatinib PBPK models are listed in Table 1. The physicochemical parameter of molecular weight was obtained from drugbank, and the oil/water partition coefficient (log P) was predicted in ALOGPS. The dissociation equilibrium constant (p*K*a) was determined in our laboratory. Also, plasma unbound fraction (f_u_) of apatinib was collected from literature (Ding et al., 2013). The blood to plasma partition coefficient (B/P) was calculated using Simcyp Simulator. The *in vivo* absorption of apatinib was determined using the Advanced Dissolution Absorption and Transit (ADAM) models, in which the gastrointestinal tract was divided into nine successive compartments. A drug could exist in several states simultaneously within each compartment. The mainly involved parameters in the ADAM setting were permeability and dissolution data. Caco-2 permeability data were used to calculate the effective permeability (P_eff_) of apatinib in humans. The *in vitro* dissolution profile of apatinib mesylate tablet was loaded to describe the *in vivo* release of the drug. The tissue to plasma partition coefficients (K_p_) of apatinib for all the major tissues in Simcyp Simulator were calculated using Poulin and Theil method with a K_p_ scalar of 0.7. The volume of distribution at steady state (V_ss_) was predicted using physicochemical properties and K_p_. Apatinib was reported to be extensively metabolized *via* CYP3A4/5 and CYP2D6 (Ding et al., 2013). The enzyme kinetics was used to describe the *in vivo* clearance of apatinib. The maximum velocity (V_max_) and Michaelis–Menten constant (K_m_) were obtained from the reported metabolism and pharmacokinetics of apatinib in humans (Ding et al., 2013).

**TABLE 1:** Summary input data for apatinib in Simcyp Simulator simulation

Parameters		Value	Source
**Physiochemical parameters**			
Molecular weight (g/mol)		397.48	drugbank
log P		3.14	ALOGPS
Compound type		Dibasic base	
p*K*a		p*K*a_1_ = 6.60 p*K*a_2_ = 5.31	In-house data
B/P		0.995	Calculated using Simcyp
f_u_		0.076	Ding et al. (2013)
**Absorption parameters**			
**ADAM model**
Caco-2 permeability (10^−6^ cm/s)		6.81	In-house data
P_eff_ (10^−4^ cm/s)		0.80	Calculated using Simcyp
**Disposition parameters**			
Full PBPK model			Poulin and Theil method
K_p_ scalar		0.7	Calculated using Simcyp
V_ss_ (L/kg)		2.684	Calculated using Simcyp
**Elimination parameters**			
Enzyme		CYP 2D6	Ding et al. (2013)
V_max_ (pmol/min/mg protein)		9.82	
K_m_ (μM)		1.41	
Enzyme		CYP 3A4	
V_max_ (pmol/min/mg protein)		39.1	
K_m_ (μM)		2.18	
Enzyme		CYP 3A5	
V_max_ (pmol/min/mg protein)		3.28	
K_m_ (μM)		1.93	

### *In vitro* Permeability Experiment

Caco-2 cells were cultured in Dulbecco’s Modified Eagle Medium (DMEM), which contained 10% fetal bovine serum, 100 U/ml penicillin G, 100 mg/ml streptomycin, 1% nonessential amino acids, and 2 mM glutamine, at the atmosphere of 37°C and 5% CO_2_. Caco-2 cells in logarithmic growth phase were seeded at a density of 4 × 10^4^/well onto a 24 Transwell plate. The DMEM was added at a volume of 200 μl for apical (AP) compartments and 1,300 μl for basolateral (BL) compartments, and changed every other day for 21 days. Then the cell layer was washed three times with pre-heated HBSS buffer, and the transepithelial electrical resistance (TEER) values were measured. Caco-2 monolayers with TEER values >350 Ω·cm^−2^ were used for permeability test.

The drug solutions of apatinib mesylate (10.0 μM), atenolol (10.0 μM), propranolol (10.0 μM), and digoxin (20.0 μM) were prepared with HBSS buffer consisting of 0.5% BSA. The pH was adjusted to 7.4 with 1 M NaOH. The Caco-2 monolayers were rinsed with Hank’s balanced salt solution before the experiment and were incubated at 37°C for 1 h. Then, the tested compound was added to the AL or BL compartments of the polycarboxylate membrane, and the cell culture plate was placed at the atmosphere of 37°C and 5% CO_2_. After 2 h of circular vibration culture, samples at a volume of 150 μl were taken from the AL and the BL compartments. The P_app_ values of compounds from AL to BL compartments and BL to AL compartments were calculated by the following formula:

$$P_{app}=\frac{\mathrm{△Q}}{△T\times A\times C_{0}}$$

A is the area of the polycarboxylate membrane and C_0_ is the initial concentration.

### Verification and Evaluation the Performance of PBPK Model

The pharmacokinetics of apatinib was simulated *via* the dynamic PBPK model. The characteristics of subjects and trial design adhered to the clinically reported study, and the simulated results were compared with the observed data (Liu et al., 2018). The fold error of the main pharmacokinetic parameters (C_max_, T_max_, and AUC) was used to assess the predictive accuracy, which referred to the ratio of the simulated to the observed values (Eq. 1). A desired fold error was between 0.5 and 2.0 (Zhang et al., 2018; Li et al., 2019).

$$fold\;error=\frac{simulated}{observed}$$ (1)

For verification of the apatinib DDI models, the DDI predictions of apatinib were performed with itraconazole (strong CYP3A4 inhibitor) and rifampin (strong CYP3A4 inducer). The characteristics of subjects and trial design were based on the clinical study, and the simulated results were compared with the observed data (Liu et al., 2018). The change of apatinib exposure was determined by AUC ratio and C_max_ ratio, which referred to the mean AUC and C_max_ in the presence to the absence of inhibitor or inducer, respectively (Eq. 2). The current consensus for successful DDI simulation is that the predicted AUC ratio or C_max_ ratio should be less than 2-fold (Zhang et al., 2018; Li et al., 2019).

$$AUC\;ratio=\frac{AUC\;with\;inhibitor\;or\;inducer}{AUC\;without\;inhibitor\;or\;inducer}$$

$$C_{max}\;ratio=\frac{C_{max}\;with\;inhibitor\;or\;inducer}{C_{max}\;without\;inhibitor\;or\;induce}$$ (2)

### Application of the PBPK Models for Evaluating the Influence of Co-Administered Possible Inhibitors and Inducers on Apatinib Exposure

The PBPK models were used to predict potential DDI risks with CYP2D6 and CYP3A4 perpetrators. For apatinib DDI simulation with moderate and strong CYP2D6 inhibitors, fluvoxamine (50 mg once daily (qd)), quinidine (200 mg qd), and paroxetine (30 mg qd) were used for the PBPK modeling. Erythromycin (250 mg four times a day (qid)) and verapamil (120 mg three times a day (tid)) were used for apatinib DDI simulation with moderate and weak CYP3A4 inhibitors. For apatinib DDI simulation with CYP3A4 inducers, carbamazepine (400 mg twice daily (bid)), efavirenz (600 mg qd), and phenytoin (300 mg qd) were used for the PBPK modeling.

Simulation of all DDI outcomes were performed using Simcyp Chinese healthy volunteers in a fasted state with 100 subjects (10 trials×10 subjects). The outline of the trial design is summarized in Table 2.

**TABLE 2:** Summary of the outline of the trial design for apatinib DDI simulation

Dosing regimen of apatinib	Co-administered drug	Dosing regimen of co-administered drug		Possible pathway of DDI	
750 mg on day 4	Fluvoxamine	50 mg qd	day 1–6	Inhibition	CYP2D6
	Quinidine	200 mg qd		
	Paroxetine	30 mg qd		
	Erythromycin	250 mg qid			CYP3A4
	Verapamil	120 mg tid			
750 mg on day 8	Carbamazepine	400 mg bid	day 1–10	Induction	CYP3A4
	efavirenz	600 mg qd			
	phenytoin	300 mg qd			

### Application of the PBPK Models for Predicting the Pharmacokinetic Changes in HIs and RIs

A single-dose DDZI potential of apatinib in HIs and RIs was evaluated by the PBPK models. The simulation was conducted in a fasted state with 100 subjects (10 trials×10 subjects) receiving an oral dose of 750 mg apatinib. Virtual populations with Child–Pugh scores A, B, and C in Simcyp in-built population library were used for simulation in mild, moderate, and severe HIs, respectively. The Simcyp in-built moderate (glomerular filtration rates (GFR) of 30–60 ml/min) and severe RI (GFR <30 ml/min) populations were used for simulation in RIs. The pathophysiological changes incorporated within the disease populations used for performing simulations were reduced liver size and kidney weight, reduced hepatic CYP expression (e.g., CYP1A2, 2C9, 2C19, 2D6, and 3A4), reduced serum albumin, α-1 acid glycoprotein levels as well as hematocrit levels, altered blood flow, and so on (Ono et al., 2017).

## Results

### Verification of the Dynamic PBPK Model for Apatinib

**FIGURE 1:**
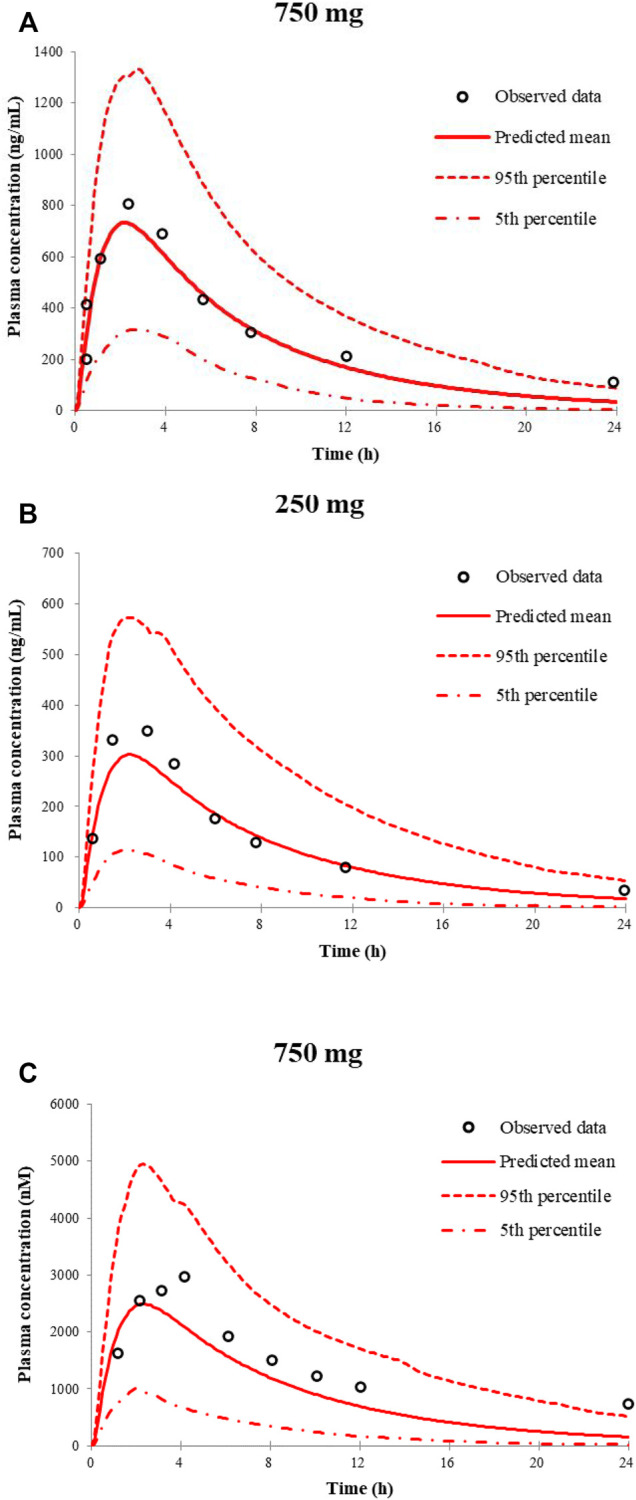
Predicted and observed mean plasma concentration–time curves of apatinib following a single oral dose of apatinib mesylate tablet in healthy volunteers **(A**,**B)** and in cancer patients **(C)**.

Figure 1 shows the simulated and observed mean plasma concentration–time curves of apatinib at 750 and 250 mg dose levels in healthy volunteers, as well as the comparison of model-predicted 750 mg dose level in Chinese healthy volunteers and the observed 750 mg dose level in cancer patients. The comparison of the model-predicted main pharmacokinetic parameters (C_max_, T_max_, and AUC) with the observed data and the calculated fold errors for the main pharmacokinetic parameters of apatinib are summarized in Table 3. The predicted mean plasma concentration–time curves of apatinib matched well with the clinically observed one. All the fold errors of C_max_, T_max_, and AUC were between 0.5 and 2.0 of the observed data, indicating a good prediction of the apatinib PBPK model. The model-predicted main pharmacokinetic parameters of apatinib in Chinese healthy volunteers were comparable with the observed one in cancer patients.

**TABLE 3:** Comparison of model-predicted main pharmacokinetic parameters (C_max_, T_max_, and AUC) to the observed data of apatinib mesylate tablet

Dose level	Parameters	Unit	Predicted	Observed	Fold error
750 mg (in Chinese healthy volunteers)	T_max_	h	2.16	3	0.72
	C_max_	ng/ml	734.76	681	1.08
	AUC	ng/ml·h	5,994.63	7,150	0.84
250 mg (in Chinese healthy volunteers)	T_max_	h	2.16	1.5	1.44
	C_max_	ng/ml	302.47	371	0.82
	AUC	ng/ml·h	2,626.4	2,940	0.89
750 mg (in Chinese cancer patients)	T_max_	h	2.28	2.9	0.79
	C_max_	nM	2,497.76	3,819	0.65
	AUC	nM·h	22,274.01	30,941	0.72

### Evaluation of the DDI Prediction for Apatinib PBPK Model

**FIGURE 2:**
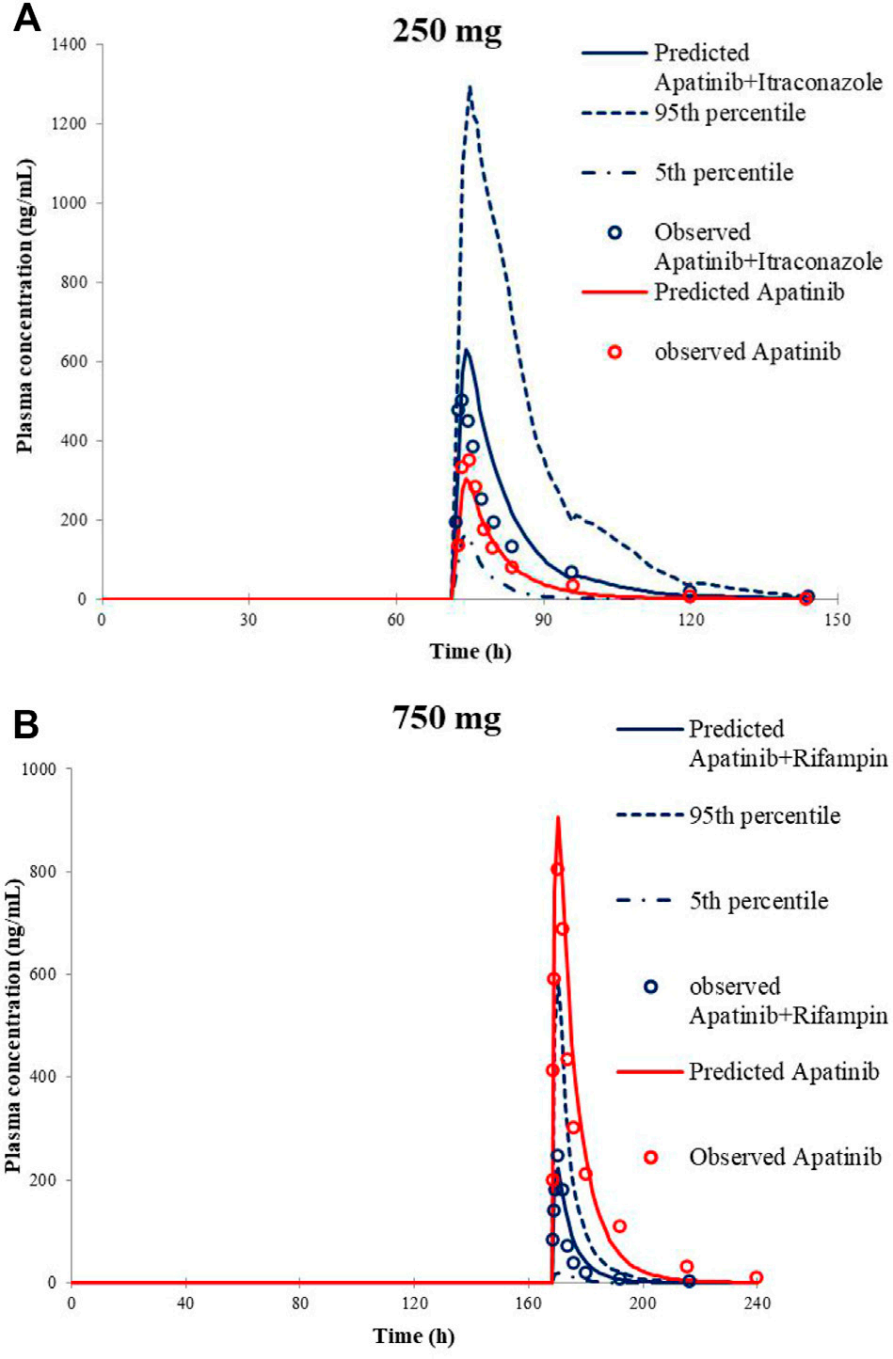
Predicted and observed mean plasma concentration–time curves of apatinib following 250 mg apatinib mesylate tablet in the presence or absence of itraconazole **(A)** and 750 mg apatinib mesylate tablet in the presence or absence of rifampin **(B)**.

The predicted along with observed mean plasma concentration–time curves of apatinib with co-administered itraconazole or rifampin are described in Figure 2. Table 4 shows the predicted and observed changes of apatinib exposure in the presence of strong inhibitor or inducer of CYP3A4, and calculated AUC ratio as well as C_max_ ratio in the presence to the absence of inhibitor or inducer, respectively. On the basis of the results, the developed PBPK model successfully simulated the DDIs of apatinib with itraconazole, and rifampin. Thus, investigation of the DDIs of apatinib could be conducted by the verified PBPK models.

**TABLE 4:** Comparison of model-predicted C_max_ and AUC to the observed one in the presence or the absence of inhibitor or inducer

Co-administered drugs	Simulated		Observed		C_max_ ratio_s_/C_max_ ratio_o_	AUC ratio_s_/AUC ratio_o_
	C_max_ ratio	AUC ratio	C_max_ ratio	AUC ratio		
With itraconazole	2.08	2.69	1.34	1.71	1.55	1.57
With rifampin	0.30	0.28	0.39	0.17	0.77	1.65

### Application of the Apatinib PBPK Model for the Evaluation of the Potential DDI Risks

**FIGURE 3:**
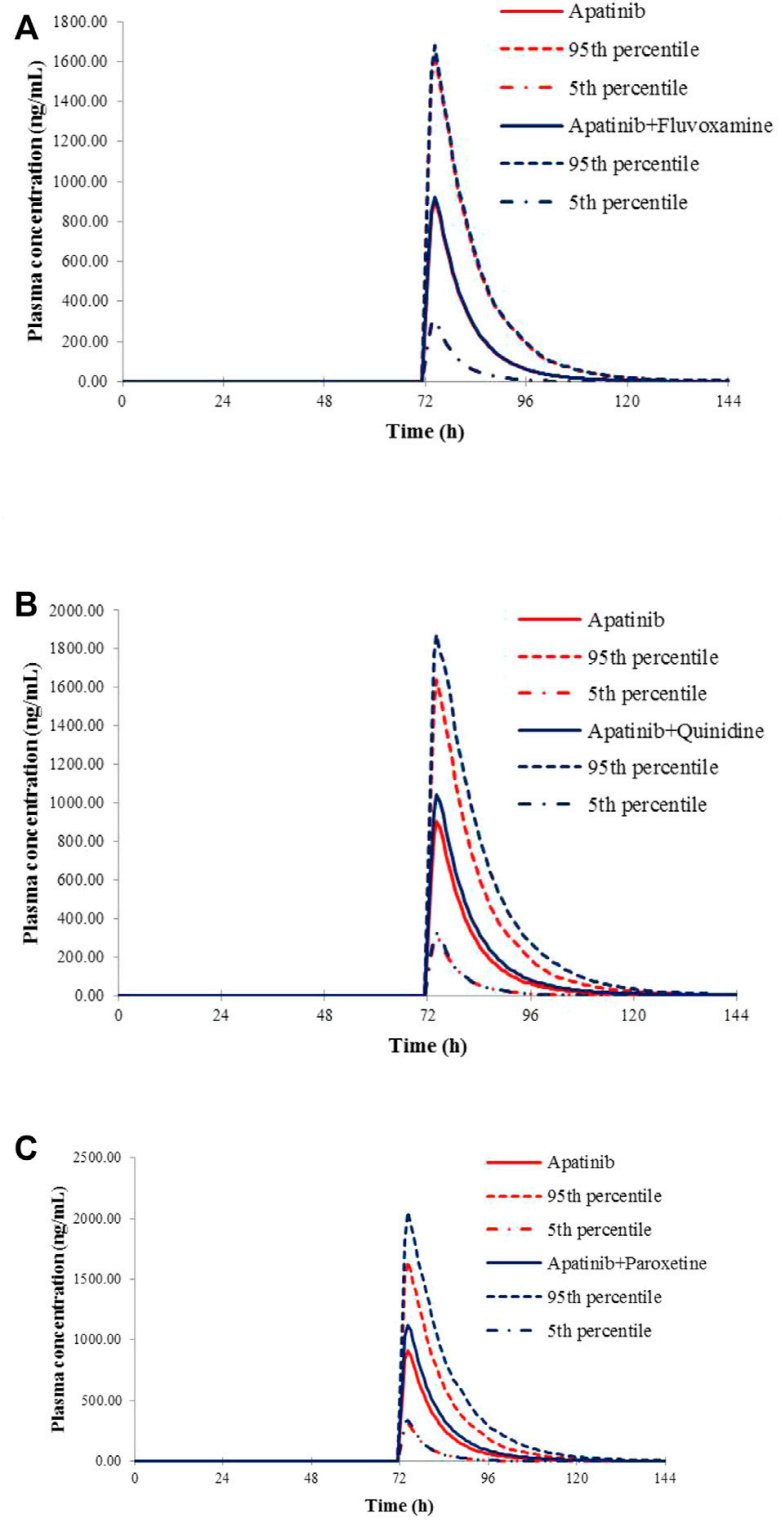
Predicted mean plasma concentration–time curves of apatinib with or without co-administration of CYP2D6 inhibitors, fluvoxamine **(A)**, quinidine **(B)**, and paroxetine **(C)**.

**FIGURE 4:**
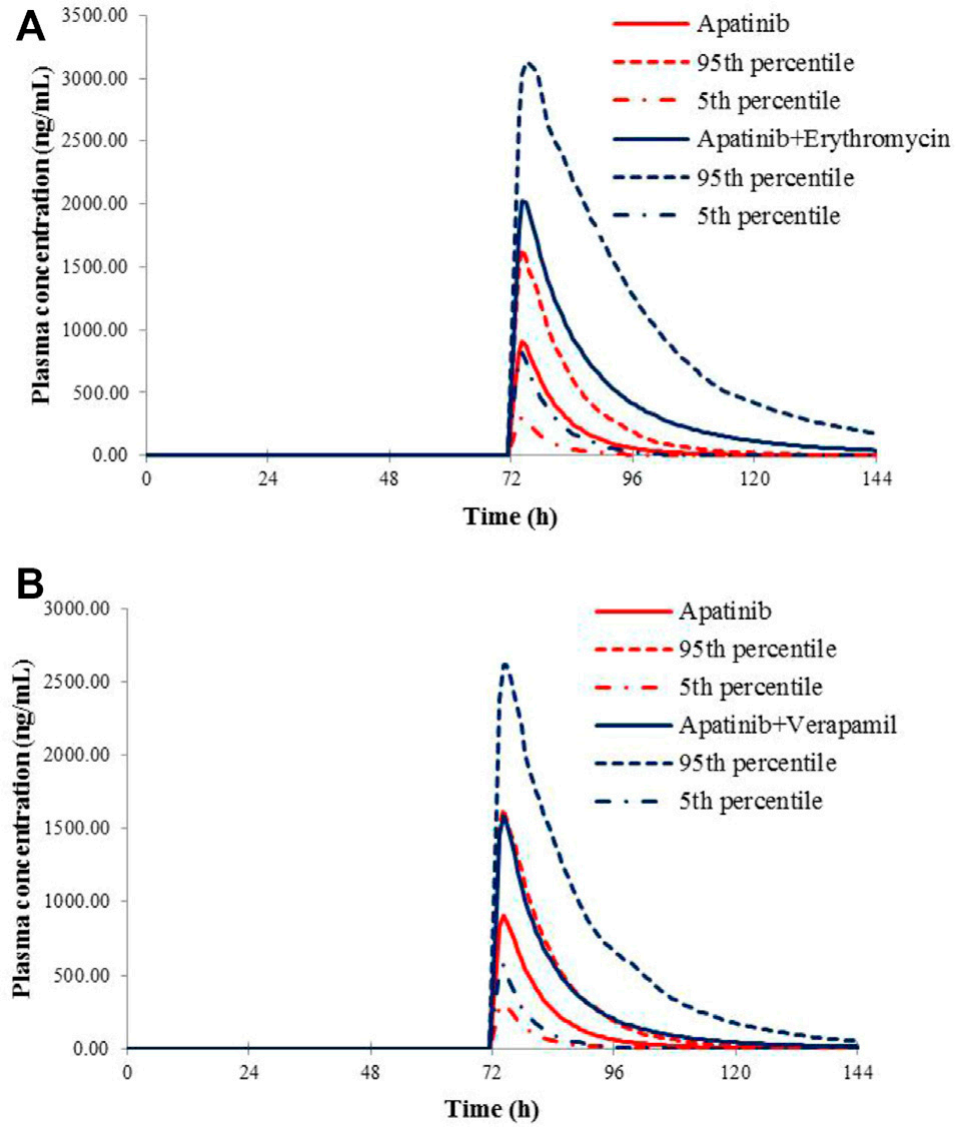
Predicted mean plasma concentration–time curves of apatinib with or without co-administration of CYP 3A4 inhibitors, erythromycin **(A)**, and verapamil **(B)**.

**FIGURE 5:**
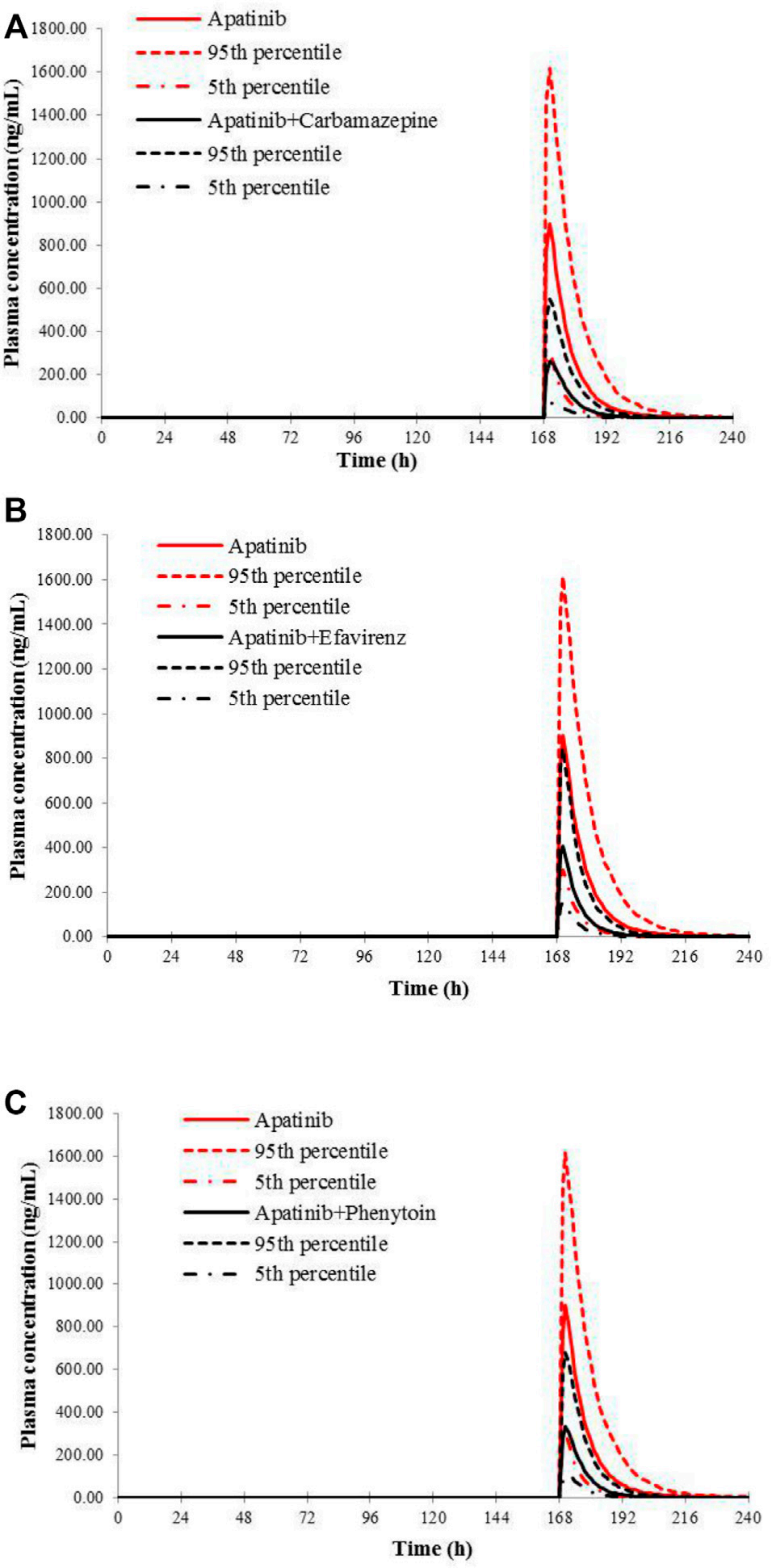
Predicted mean plasma concentration–time curves of apatinib with or without co-administration of CYP 3A4 inducers, carbamazepine **(A)**, efavirenz **(B)**, and phenytoin **(C)**.

The verified PBPK models of apatinib were applied to evaluate the potential DDI risks with moderate and weak CYP3A4 inhibitors, strong CYP2D6 inhibitors, and the CYP3A4 inducers at therapeutic doses. The trial design was based on the clinical reports shown in Table 2. The DDI outcomes are depicted in Figures 3–5, and the calculated ratio of C_max_ or AUC is shown in Table 5. The exposure change (C_max_ or AUC) by over 2-fold was considered to be significant in the DDI simulations. In this context, the exposure of apatinib was obviously affected by co-administration of erythromycin, verapamil, carbamazepine, efavirenz, and phenytoin, respectively. A moderate increase of apatinib exposure (C_max_ or AUC ratio between 1.25 and 2.0) was found with paroxetine. It appeared that moderate CYP2D6 inhibitor fluvoxamine and quinidine had negligible influence on the exposure of apatinib at therapeutic dose.

**TABLE 5:** Simulated exposure changes of apatinib with co-administration of possible inhibitors or inducers

Dosage regime of apatinib	Co-administered inhibitors or inducers	Dosage regime of co-administered drugs		Simulated	
				C_max_ ratio	AUC ratio
750 mg on day 4	Fluvoxamine	50 mg qd	Days 1–6	1.02	1.02
	Quinidine	200 mg qd		1.15	1.21
	Paroxetine	30 mg qd		1.23	1.29
	Erythromycin	250 mg qid		2.23	3.60
	Verapamil	120 mg tid		1.76	2.17
750 mg on day 8	Carbamazepine	400 mg bid	Days 1–10	0.29	0.30
	Efavirenz	600 mg qd		0.45	0.36
	Phenytoin	300 mg qd		0.37	0.34

### Application of the Apatinib PBPK Model for Evaluation of the Potential DDZI Risks

**FIGURE 6:**
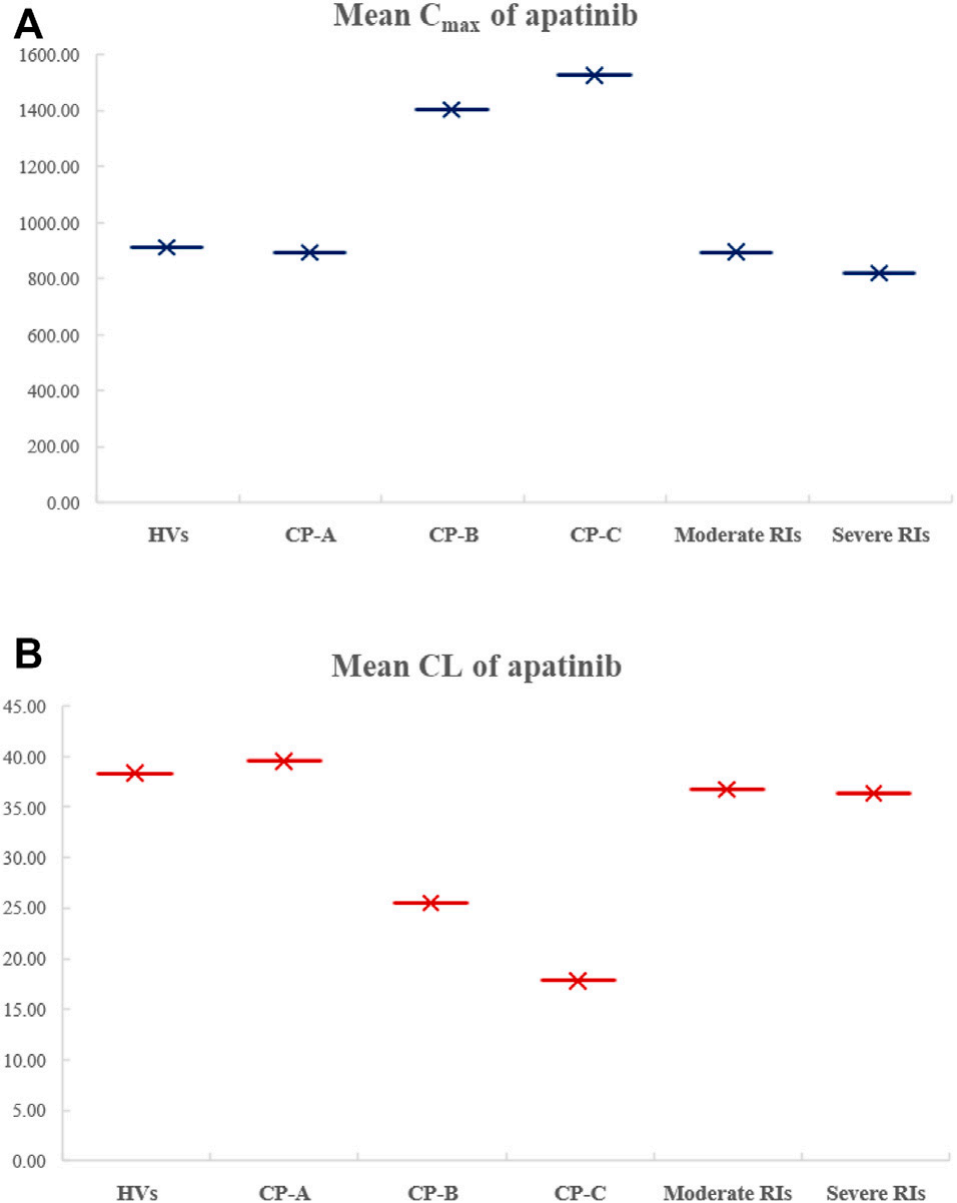
Comparison of the simulated mean C_max_ **(A)** and CL **(B)** of apatinib in HVs, in HIs with Child–Pugh A, B, or C, as well as in moderate and severe RIs.

The virtual populations of Chinese healthy volunteers (HVs), HIs with Child–Pugh A, B, or C, and moderate and severe RIs in Simcyp in-built population library received an oral dose of 750 mg apatinib mesylate tablet. The simulated mean plasma concentration–time curves are depicted in Figure 6. As shown in Table 6, the predicted C_max_ ratio and AUC ratio were 1.02 and 1.09 for Child–Pugh A, 1.54 and 2.25 for Child–Pugh B, and 1.67 and 3.04 for Child–Pugh C, respectively, in HIs. The predicted C_max_ ratio and AUC ratio were 1.02 and 1.06 for moderate RIs, and 1.11 and 1.01 for severe RIs, respectively. The impact of RIs was negligible on the exposure of apatinib at therapeutic dose.

**TABLE 6:** Simulated exposure changes of apatinib in patients with HI and RI

Subjects	C_max_ (ng/ml)	AUC (ng/ml·h)	Ratio	
			R_Cmax_	R_AUC_
HVs	911.56	7,934.66	/	
HI CP-A	892.54	8,641.86	1.02	1.09
HI CP-B	1,402.36	17,880.77	1.54	2.25
HI CP-C	1,524.33	24,141.57	1.67	3.04
Moderate RIs	894.98	8,449.86	1.02	1.06
Severe RIs	820.29	8,050.40	1.11	1.01

## Discussion

In the present study, we first developed a PBPK model for apatinib by incorporating both *in vitro* and *in vivo* data. The pharmacokinetics of apatinib at an oral dose level of 750 and 250 mg in healthy volunteers, as well as 750 mg dose level in cancer patients, were predicted. The results showed that the pharmacokinetic profiles of apatinib matched well with the clinically observed one. Baker et al. have reported that age, sex, and body size have negligible influence on the CYP3A activity in 134 cancer patients (Baker et al., 2004). Consistently, midazolam, a CYP3A probe substrate, was predicted to have comparable exposure between healthy volunteers and cancer patients using PBPK modeling (Cheeti et al., 2013). The PBPK model developed by Chiho et al. captured comparable pharmacokinetics of bosutinib in healthy volunteers with cancer patients as well (Ono et al., 2017). Therefore, the Simcyp default “Chinese Healthy Volunteers” population file was used both in simulation with healthy volunteers and cancer patients. Our study also demonstrated the similar simulation in these two populations. Afterwards, the validation of the apatinib DDI model was conducted using the reported clinical study, which showed the successful simulation of the exposure changes of apatinib in the presence or absence of itraconazole and rifampicin (Liu et al., 2018).

Then, the potential risks of DDI and DDZI of apatinib were estimated using the verified PBPK model. Owing to that cancer patients are frequently treated with multiple medications, the potential DDI risks of apatinib co-administered with other drugs must be determined. Apatinib was reported to be metabolized primarily *via* CYP3A4/5 and CYP2D6 (Ding et al., 2013). Therefore, the verified PBPK model was applied to evaluate the potential DDI risks involving CYP3A4 and CYP2D6-mediated metabolizing pathways. Erythromycin and verapamil were moderate CYP3A4 inhibitors (Zhou, 2008; Yamazaki et al., 2015). In our simulations, apatinib exposure was obviously increased in subjects concomitantly treated with erythromycin and verapamil. The predicted AUC of DDI ratio was 3.60 and 2.17 with co-administration of erythromycin and verapamil, respectively. The presence of paroxetine, a strong CYP2D6 inhibitor, could also lead to a moderate increase of the exposure of apatinib by 1.29-fold. The simulated result was also consistent with the fact that the metabolism of apatinib *via* CYP3A4 was much higher than that of CYP2D6. Thus, attention should be paid to the co-administration of apatinib with moderate CYP3A4 inhibitors and the corresponding dose adjustment in clinic. In the DDI simulation with CYP3A4 inducers, the predicted apatinib exposure decreased by 71.8, 61.6, and 63.9% in the presence of carbamazepine, efavirenz, and phenytoin, respectively, indicating that we should be careful about the pharmacokinetic interactions of apatinib with CYP3A4 inducers, which is likely to impair the efficacy.

It has been reported that cirrhosis could decrease gastrointestinal absorption and reduce the expression of drug-metabolizing enzymes as well as transporters (Verbeeck, 2008). The renal impairment could cause decreases in renal clearance along with other changes such as activity of drug-metabolizing enzyme, especially in CYP3A4 and reduced plasma protein binding (Yeung et al., 2014). Thus, the DDZI risks should be assessed in HIs and RIs for appropriate dosing recommendations. In the DDZI simulation with HIs, the AUC of apatinib was significantly increased by 2.25-fold and 3.04-fold for Child–Pugh B and Child–Pugh C, respectively, with slight but consistent decrease of C_max_ by 1.54- and 1.67-fold, respectively. The results were in accordance with the alteration of absorption and the decrease in CYP enzyme-mediated clearance of apatinib due to decreased enzyme abundances in patients with HIs. The model-predicted AUC ratios in moderate and severe RIs were 1.06 and 1.01, respectively, suggesting the negligible impact of renal impairment on the exposure of apatinib. In view of the challenges to conduct various DDI and DDZI studies in cancer patients, the PBPK model would be of great benefit to quantitatively predict pharmacokinetic changes under different circumstances which might be difficult to evaluate clinically, so as to avoid some DDI and DDZI risks in advance.

However, some limitations in this study warrant further discussion. First, the present study focused on the pharmacokinetic changes of apatinib as a victim drug; the impact of apatinib as perpetrators on other drugs being mainly metabolized *via* CYP3A4 or CYP2D6 has not been evaluated. It has been demonstrated that apatinib inhibited the metabolism of gefitinib *in vitro* and *in vivo via* CYP2D6 and CYP3A4 (Wang et al., 2021). Zhu et al. have investigated the potential of apatinib as a perpetrator on the pharmacokinetics of nifedipine and warfarin in advanced solid tumor patients. The results indicated that concomitant administration of apatinib led to an obvious increase to nifedipine and warfarin exposure (Zhu et al., 2020). Due to the risk of pharmacokinetic DDI based on enzyme inhibition by apatinib, a dynamic PBPK model is needed for further dosing recommendations when apatinib is co-administered with CYP3A4, CYP2D6, or CYP2C9 substrates. Second, we only evaluated the DDZI outcomes at a single dose level; the multiple-dose DDZIs were not incorporated in the simulation, although the comparable changes of exposures in HIs between single- and multiple-dose administrations were found in some drugs such as bosutinib (Ono et al., 2017). Third, the model-predicted impacts of HIs and RIs on the pharmacokinetics of apatinib required further clinical verification, considering potential physiological changes resulted from impaired hepatic or renal function. There exist considerable challenges to accurately incorporate the complex physiological changes into PBPK models due to the changes of hepatic architecture in HIs. As a result, bosutinib exposures could not be well predicted in HIs compared with the clinical observed data by Chiho et al. (Ono et al., 2017). The apatinib PBPK model is expected to be further refined using the post-marketing observed data in HIs and RIs, and therefore more adequately evaluating the potential DDZI risks.

All in all, the present study showed that the verified PBPK model of apatinib could provide mechanistic insights for further understanding the potential risk of DDIs and DDZIs, providing the reasonable dosing recommendations.

## Conclusion

In the present study, we have developed and verified a dynamic PBPK model of apatinib based on the currently available data. The models can be used to predict apatinib exposures in the single-dose DDIs with other CYP inhibitors and DDZIs in HIs or RIs. The DDI prediction indicated significant changes (2–4-fold) in apatinib exposures with moderate CYP3A4 inhibitors or CYP3A4 inducers. A moderate increase of apatinib exposure (1.25–2-fold) was found with strong CYP2D6 inhibitor. Clinicians should be cautious about the necessary dose adjustment of apatinib being used with these perpetrators. In the DDZI simulation with HIs, the AUC of apatinib was significantly increased by 2.25-fold and 3.04-fold for Child–Pugh B and Child–Pugh C, respectively, with slight but consistent decrease of C_max_ by 1.54- and 1.67-fold, respectively. The DDZI prediction suggested a negligible increase in apatinib exposures in RIs. The PBPK models could be of great benefit to give some recommendations in DDI and DDZI risk assessments, and replace some clinical studies as well. The verified PBPK model could provide a rough understanding of the extrinsic and intrinsic factors on the exposure of apatinib.

## Data Availability

The original contributions presented in the study are included in the article/Supplementary Material; further inquiries can be directed to the corresponding authors.
